# The Role of Gut Microbiota in Gastrointestinal Tract Cancers

**DOI:** 10.1007/s00005-021-00641-6

**Published:** 2022-02-03

**Authors:** Marta Grochowska, Karol Perlejewski, Tomasz Laskus, Marek Radkowski

**Affiliations:** 1 Department of Immunopathology, Infectious and Parasitic Diseases, Medical University of Warsaw, Warsaw, Poland; 2 Department of Adult Infectious Diseases, Medical University of Warsaw, Warsaw, Poland

**Keywords:** Microbiota, Gastrointestinal tract, Cancer, Oral, Esophageal, Gastric, Pancreatic, Hepatocellular, Colorectal

## Abstract

Disturbances in gastrointestinal (GI) microbiota could play a significant role in the development of GI cancers, but the underlying mechanisms remain largely unclear. While some bacteria seem to facilitate carcinogenesis, others appear to be protective. So far only one bacterium (*Helicobacter pylori*) has been classified by the International Agency for Cancer Research as carcinogenic in humans but many other are the subject of intense research. Most studies on the role of microbiota in GI tract oncogenesis focus on pancreatic and colorectal cancers with the following three species: *Helicobacter pylori, Escherichia coli*, and *Porphyromonas gingivalis* as likely causative factors. This review summarizes the role of bacteria in GI tract oncogenesis.

## Introduction

Cancer is currently a major world-wide health problem: it is estimated that approximately 18.1 million new cancer cases and 9.6 million cancer-related deaths occurred in 2018 alone, and there is a 20% risk of developing cancer before turning 75 years old, and 10% risk of dying from it (Ferlay et al. 2019). Infectious agents are estimated to be responsible for 17.8% of all cancers; specifically, viruses could be responsible for 12.1%, bacteria for 5.6% and helminths for 0.1% of cases (de Martel et al. 2012; Parkin 2006). In contrast to viral-related oncogenesis, very little is known about the role of bacteria in cancer development; however, it is likely that understanding the long-term effects of changes in gastrointestinal (GI) microbiota composition could facilitate the development of cancer preventive strategies (Chang and Parsonnet 2010). Bacteria may also be involved in carcinogenesis indirectly by modulating local and systemic immune responses, which are crucial for the development of the GI tract cancers (Velikova et al. 2021).

The human GI microbiota, defined as the ecological community of microorganisms (Wei et al. 2019), plays a plethora of beneficial roles including detoxification, reduction of inflammation, and balancing of host cell proliferation and growth (Garrett 2015). The microbiota colonizes GI tract shortly after birth and remains for the whole life, but it can undergo dynamic changes related to such factors as diet, environmental stressors, lifestyle, antibiotics, and other drugs (Wei et al. 2019). Bacteria living in the human gut achieve the highest documented cell concentration for any ecosystem 10^11^–10^12^ per mL (Hu et al. 2016) and Bacteroidetes and Firmicutes are the two dominant phyla in the stool microbiome (Leite et al. 2020). Altogether, 11 microorganisms were named by the International Agency for Cancer Research as carcinogenic to humans including only one bacterium (*Helicobacter pylori)* (de Martel et al. 2012; Garrett 2015). Despite the fact that colonization by these microbes is widespread, only a minority of people develop cancer in their lifetime due to multifactorial nature of oncogenesis (Garrett 2015).

GI microbiota varies between individuals, but the most common phyla in healthy people are Firmicutes and Bacteroidetes (Lloyd-Price et al. 2016; Lukovic et al. 2019). The GI microbiota, by its interaction with the host, plays an important role in maintaining health but it can also facilitate disease development (Hold and Hansen 2019). In animal models of gut dysbiosis, defined as a shift in microbial composition and function (Fond et al. 2015), it may affect such organs as the brain, lungs, and kidneys (Lukovic et al. 2019). Due to the high prevalence of GI cancers, relatively well-known effects of gut microbiota composition on GI tract functioning and easy access to fecal sampling, the most studies on the bacterial role in oncogenesis relate to the gut.

Gut microbes may not only play a role in stimulating carcinogenesis, but also in cancer prevention, and may even modulate cancer treatment effectiveness including, chemo-, immuno-, and radiotherapy (Kashyap et al. 2021). The potential role of gut microbiota in cancer development is supported by findings that fecal microbiota transplantation from mice with chemically induced colorectal cancer to germ-free mice markedly increases susceptibility of the latter to colonic tumorigenesis (Baxter et al. 2014).

Importantly, the effects of microbiota on cancer development may be contradictory as some bacteria were reported to facilitate, while others seem to oppose carcinogenesis in the GI tract (Garrett 2015). For example, Baxter et al. (2014) found in mice models that several members of the *Bacteroidales* (*Bacteroides*, *Parabacteroides*, *Alistipes*, and *Porphyromonodaceae*) correlated positively with tumor development, while members of the *Clostridiales*, especially *Clostridium* Group XIVa, were associated with decreased cancer risk, probably due to the production of butyrate, which has anti-inflammatory and anti-tumorigenic properties (Pryde et al. 2002).

Multiple mechanisms were proposed to explain bacteria-related oncogenesis and it seems that bacteria can affect both the initiation stage of tumour development as well as facilitate its further growth. Obviously, microbiota could promote cancer development and progression simultaneously, as demonstrated in a colorectal (CRC) mouse model in which gut microbiota changes, which occurred during tumorigenesis, supported increased tumorigenic process in the later stages (Baxter et al. 2014).

It was proposed that carcinogenicity is mainly attributed to microbial dysbiosis (Meng et al. 2018). Two of the best characterized mechanisms of bacterial-related carcinogenesis are chronic inflammation and production of toxic metabolites. Inflammation is a well-established risk factor for many cancers, including CRC (Balkwill and Mantovani 2001). The evidence for an important role of microbiota in regulating immune response is provided by interleukin (IL)-10 knock out mice (*Il10*^*−/−*^*)*, which develop spontaneous colitis due to microbial-induced activation of effector T cells (Kuhn et al. 1993). In the study by Uronis et al. (2009), germ-free Il10^−/−^ mice were bred in specific pathogen-free conditions for 20 weeks and then exposed to azoxymethane, which is a carcinogenic and neurotoxic chemical compound. In this experiment 62% of Il10^−/−^ animals developed colon tumors compared to only 20% of the wild-type mice. Various mechanisms by which GI microbiota could contribute to GI cancers are listed in Table 1.

**Table 1:** Mechanisms by which GI microbiota could contribute to GI cancers

Mechanism of action	Example	References
Chronic inflammation	IL-1, IL-6, TNF-α, IL-23, and reactive oxygen species could promote CRC development by enhancing DNA damage in epithelial cells; these cytokines activate NF-κB, Wnt signaling and mitogen-activated protein kinases pathways and cause apoptosis inhibition and increased oxidative stress	Arthur et al. (2012) and Klampfer (2011)
	IL-6 and IL-11 could sensitize signal transducer and activator STAT3, which plays an important role in transforming epithelial cells	Putoczki et al. (2013)
	Increasing intestinal permeability allows for leakage into circulation of antigens which activate the immune system	Critchfield et al. (2011), Elson and Alexander (2015), Fond et al. (2015) and Karakula-Juchnowicz et al. (2016)
	Since the innate immune system can recognize such bacterial components as LPS, flagellin and peptidoglycan, gut microbiota dysbiosis can influence innate and adaptive immune responses involved in the tumor formation process	Meng et al. (2018), Palm et al. (2015) and Vijay-Kumar and Gewirtz (2009)
Bacterial metabolites	Obesity in mice can result in the increased growth of *Clostridia* spp. producing deoxycholic acid, which can cause DNA damage and thus promote development of HCC	Yoshimoto et al. (2013) and Raza et al. (2019)
	Induction of such hormones as somatostatin or gastrin, which increase epithelial cell growth, may affect the balance between host cell proliferation and death favouring the former, while production of toxic carcinogenic metabolites by bacteria may affect various cells and consequently lead to cell transformation	Chang and Parsonnet (2010) and Garrett (2015)
	Colibactin produced by *Bacillus fragilis* and *E. coli* and cytolethal distending toxin made by *E. coli, Salmonella typhi* and *H. pylori* facilitate carcinogenesis by causing transient DNA damage, which is followed by cell divisions with incomplete DNA repair resulting in anaphase bridges and chromosome aberrations	Cuevas-Ramos et al. (2010) and Raza et al. (2019)
	Horizontal gene transfer could result in transmission of oncogenes between pathogenic and commensal bacteria	Stecher et al. (2012)
	The gut dysbiosis may result in the increase of such bacterial metabolites as acetaldehyde, secondary bile acid, and glucuronic acid, which together with enzymes and immune factors activated by microbes were proposed as potential biomarkers for GI cancers	Kashyap et al. (2021)
Epigenetic modifications	Gastritis with *H. pylori* infection is associated with hypermethylation of the promoter region of E-cadherin, the DNMT MGMT, the Wnt inhibitor WIF1, and the MLH1 gene Fusobacterium nucleatum was correlated with wild-type tumor suppressor TP53, methylation of the mismatch repair gene hMLH1, genomic hypermutation, and mutation of the chromatin remodelers CHD7/8	Kawanaka et al. (2016) and Tahara et al. (2014)
	Histone modifications: bacterial presence resulted in changes in histone acetylation in the proximal colon of wild-type mice	Krautkramer et al. (2016)
	In germ-free mice reconstituted with normal mouse microbiota or with *E. coli*, the lncRNA expression profiles are affected by gut flora	Liang et al. (2015)

*IL* interleukin, *CRC* colorectal cancer, *TNF-α* tumor necrosis factor-α, *NF-κB* nuclear factor kappa-light-chain-enhancer of activated B cells, *LPS* lipopolysaccharides, *HCC* hepatocellular carcinoma, *GI* gastrointestinal, *lncRNA* long non-coding RNA, *STAT* signal transducers and activator of transcription, *DNMT* DNA methyltransferase, *lncRNA* long non-coding RNA

Research on the role of specific bacteria and interactions between the host and microbiota in oncogenesis is progressing rapidly and is likely to provide new opportunities for cancer prevention and therapy (Garrett 2015). This review summarizes the current knowledge on the bacterial role in GI oncogenesis.

Comprehensive summary of the relationship between specific bacteria and GI tract cancers including short characteristics of bacteria and possible mechanism of carcinogenesis are presented in Table 2.

**Table 2:** Association of specific bacteria with gastrointestinal tract cancers (in alphabetical order)

	Name	Characteristics	Type of cancer	Comments and mechanisms of carcinogenesis	References
*Human studies*					
1	*Bacteroides fragilis*	Anaerobic, Gram-negative, rod-shaped bacterium	CRC	1. *B. fragilis* detected in biofilms coating human CRC and precancerous colonic lesions	Dejea et al. (2018)
				2. Production of toxins, breakdown of E-cadherin	Boleij et al. (2015)
2	Fusobacterium (genus)	Anaerobic, Gram-negative, non-sporeforming bacteria	Pancreatic cancer, colon adenomas	1. High number of *Fusobacteria* correlates with decreased risk of pancreatic cancer	Fan et al. (2018)
				2. *F. nucleatum* was detected in some carcinoma tissue, e.g. CRC	Mima et al. (2015)
				3. *Fusobacterium* was increased in a subset of human colon adenomas	Kostic et al. (2012)
3	*Helicobacter pylori*: the only bacteria listed as oncogenic factor by IARC Working Group on the Evaluation of Carcinogenic Risks to Humans (1994)	Microaerophilic, Gram-negative, a spiral bacterium. It colonizes 50% human and could be responsible for over 60% of stomach cancers	Gastric cancer, PDAC, esophageal adenocarcinoma	1. *H. pylori* likely a risk factor for PDAC	Wei et al. (2019)
				2. Risk factor of gastric carcinogenesis, but likely to lower the risk of esophageal adenocarcinoma (organ-specific effects of the bacterial microbiota in carcinogenesis)	Islami and Kamangar (2008) and Peek and Blaser (2002) Schwabe and Jobin (2013)
4	Other *Helicobacter spp.*	Microaerophilic, Gram-negative	Biliary cancers	1. *H. bilis* and *H. hepaticus* can colonize the gallbladder and are likely to cause biliary cancers	Chang and Parsonnet (2010) and Solnick and Schauer (2001)
				2. Frequent presence of *H. spp.* in patients with biliary tract cancer compared to healthy controls and patients with other biliary tract diseases (gallstones or cholecystitis)	de Martel et al. (2009)
5	*Neisseria spp.* (*N. meningitidis* and *N. gonorrhoeae*)	Obligately aerobic, Gram-negative bacteria, which colonize the mucosal surface in animals and humans	Tongue, pharyngeal, gastric, or colorectal cancer	*Neisseria spp.* are increased in salivary samples in patients presenting with tongue/pharyngeal, gastric, or colorectal cancer. Production of acetaldehyde by *Neisseria* correlates with increased risk of developing of GI cancers	Kageyama et al. (2019) and Salaspuro (2003)
6	*Porphyromonas gingivalis*	Anaerobic, a Gram-negative, rod-shaped	Digestive tract cancers	The increased presence of *P. gingivalis* in saliva was reported in patients with digestive tract cancers, but the exact mechanisms of carcinogenesis are unknown	Kageyama et al. (2019)
7	*Salmonella typhi*	Facultative anaerobes, Gram-negative, rod-shaped	Gall bladder cancer	Chronic typhoid carriage is the risk factor	Nath et al. (1997)
8	*Streptococcus gallolyticus*	Facultative anaerobes, Gram-positive, catalase-negative cocci	CRC	Promotes inflammation by IL-1, IL-8 and COX-2	Abdulamir et al. (2010)
*Animal studies*					
1	*Citrobacter rodentium*	Facultative anaerobic, Gram-negative	Colon cancer	Inoculation of mice with *C. rodentium* promoted mucosal hyperplasia and colon cancer	Barthold (1980) and Chang and Parsonnet (2010)
2	*Escherichia coli*	Facultative anaerobic, Gram-negative, rod-shaped, coliform bacterium	CRC	1. *E. coli* pks^+^ promotes tumorigenesis in mouse CRC models (production of colibactin responsible for mutagenic DNA damage in colonic epithelial cells)	Arthur et al. (2012) and Wilson et al. (2019)
				2. Colonization with *E. coli* was sufficient to induce tumorigenesis in GF mice. Deletion of pks decreases tumor invasion	Arthur et al. (2012)
3	Fusobacterium (genus)	Anaerobic, Gram-negative, non-sporeforming bacteria	Pancreatic cancer, colon adenomas	1. *Fusobacterium* potentiates intestinal tumorigenesis in mice	Kostic et al. (2013)
				2. The pro-oncogenic role of *Fusobacterium* was shown in mice models. Colonization by these bacteria resulted in faster tumor cell growth	Kostic et al. (2013) and Yang et al. (2017)

*GF* germ-free, *E. coli pks*^+^ *Escherichia coli* strains harboring polyketide synthase (*pks)* genomic island, *CRC* colorectal cancer, *PDAC* pancreatic ductal adenocarcinoma, *IL* interleukin, *COX-2* cyclooxygenase-2, *IACR* International Agency for Cancer Research

## Gut Microbiota in GI Cancer

### Oral Cancer

Oral cancer is one of the most prevalent cancers globally (Zhang et al. 2019b) and its most common form (> 90%) is the squamous cell carcinoma (OSCC) (Kademani 2007). Oral microbiota in patients with OSCC are characterized by the increased prevalence of anaerobic and acid-resistant bacteria (*Porphyromonas gingivalis*, *Streptococcus mitis*, *Fusobacterium*), Firmicutes (mainly *Streptococcus*), and Actinobacteria (mainly *Rothia*) (Hooper et al. 2006, 2007).

Zhang et al. (2019b) examined microbiota composition in various stages of OSCC in three different types of samples: neoplastic tissue collected during surgery, saliva, and mouthwash. The study revealed significant differences between the samples in bacterial quantity and diversity: In particular *Proteobacteria* were elevated in the cancer tissue (predominant taxa *Acinetobacter* and *Fusobacterium*), while *Firmicutes* predominated in saliva and mouthwash (predominant taxa *Streptococcus* and *Prevotella*). Interestingly, *Acinetobacter* and *Fusobacterium,* which were enriched in the neoplastic tissue, remained increased in the late stage of OSCC facilitating cancer progression by their ability to cause local inflammation. Zhang et al. (2019b) performed a series of functional analyses demonstrating that microbiota might be involved in lipopolysaccharides (LPS) synthesis and escape of host cell cycle arrest which are potential risk factors for OSCC. Importantly, LPS was described as an effector facilitating transformation of oral epithelial cells into cancer cells (Gholizadeh et al. 2017). These authors found that microbiota found in the saliva damage the environment by penetrating cells and secreting toxins (Zhang et al. 2019b). It was also reported that *F. nucleatum* infections may cause cancer through their effect on MMP9 pathways and upregulation of cytokines such as tumor necrosis factor (TNF)-α, IL-1β, and IL-6 (Whitmore and Lamont 2014).

### Esophageal Cancer

Esophageal cancer is the eighth most commonly diagnosed cancer worldwide (Parkin et al. 2005) and esophageal adenocarcinoma (EAC) accounts for more than 60% of esophageal cancers in the United States (Jain and Dhingra 2017). The only established precursor of EAC is Barrett’s esophagus (BE) (Lopetuso et al. 2020). Normal esophagus flora consist mainly of Firmicutes, especially *Streptococcus* (Baba et al. 2017), but chronic inflammation associated with gastroesophageal reflux disease may result in the increase of Gram-negative organisms such as Prevotella and Fusobacterium (Yang et al. 2009). In turn, LPS of these bacteria may activate the innate immune system facilitating the development of EAC through inflammatory cytokines IL-8 and TNF-*α* (Abdel-Latif et al. 2004; O'Riordan et al. 2005).

Lopetuso et al. (2020) found that BE and EAC patients have higher number of Operational Taxonomic Units and biodiversity when compared to healthy controls. They also observed a progressive reduction of Firmicutes to Bacteroidetes ratio during transition from BE to EAC and an increase of Leptotrichia, Veillonella and Prevotella, which are considered to be pro-oncogenic (Bundgaard-Nielsen et al. 2019; Castano-Rodriguez et al. 2017; Geng et al. 2014; Guerrero-Preston et al. 2016).

There are contradictory reports regarding the association between EAC and *H. pylori* infection. On the one hand, it was found that *H. pylori* can protect against EAC by decreasing gastric acid production (Bonde et al. 2021). On the other, Bonde et al. (2021) reported that *H. pylori* infection may dysregulate micro RNAs expression and subsequently modify intestinal metaplasia factors such as caudal-type homeobox 2 and cyclooxygenase-2.

Interestingly, the role of Campylobacter in EAC progression may mimic that of *H. pylori* in gastric cancer (Baba et al. 2017), and colonization by this bacteria results in increased expression of cancerogenic IL-18 (Blackett et al. 2013). In the esophagojejunostomy rat model, the antibiotic treatment resulted in the reduction of Lactobacillales and increase of Clostridium, but these shifts in the esophageal microbiome did not affect the incidence of EAC (Sawada et al. 2016).

### Primary Gastric Lymphomas

Primary gastric lymphomas constitute approximately 2–8% of all gastric tumors and one type in particular – marginal zone lymphoma of mucosa-associated lymphoid tissue (MALT) has become the focus of extensive microbiota analysis (Zullo et al. 2014). The latter lymphoma is characterized by activation of B and T helper cells, which are specifically reactive to *H. pylori* antigens (Wotherspoon et al. 1993). It was shown that anti-*H. pylori* treatment can prevent the development of gastric cancer and it also inhibits the progression of some precancerous lesions in humans (Correa et al. 2000; de Vries et al. 2009), and can also stop gastric cancer progression in mice (Chang and Parsonnet 2010Lee et al. 2008; Romero-Gallo et al. 2008). Antibiotics are effective in the early, but not advanced, stage of gastric cancer, although deferred therapy may still positively affect histological abnormalities in mice (Chang and Parsonnet 2010). It is worth noting some epidemiological studies have demonstrated that higher life standards and improved levels of hygiene, while decreasing the prevalence of *H. pylori* infection, do not affect the incidence of gastric cancer (de Martel et al. 2012).

*H. pylori* is likely to be a factor in the cascade leading to gastric adenocarcinoma (GAC), but infection alone is not sufficient (Kumar et al. 2020; Wang et al. 2014). Kumar et al. (2020) analyzed 371,813 veterans infected with *H. pylori* and found that successful antibiotic treatment decreased gastric cancer risk. The study also found significantly higher risks of gastric cancer were found amongracial ethnic minorities and smokers. Conversely, Nguyen et al. (2020) studied 91 patients with gastric adenocarcinoma and found that the prevalence of *H. pylori* infection was low and decreasing over time, which suggests that there are other important factors apart from *H. pylori* involved in the pathogenesis of GAC.

Early-stage immunoproliferative small intestinal disease and gastric MALT lymphoma share some histopathological features and both respond to antibiotics, which suggests a possible role of bacteria in their pathogenesis (Lecuit et al. 2004). Analysis of tissue specimens obtained from gastric, duodenal, and jejunal biopsies of patients with immunoproliferative small intestinal disease before and after antibiotic therapy suggest some role of *Campylobacter jejuni* (Lecuit et al. 2004). Thus, *C. jejuni* was detected in the biopsy samples of the small intestine by fluorescence in situ hybridization and immunohistochemical staining and its eradication by antibacterial therapy resulted in disease remission. Importantly, the 16S analysis of biopsy specimens from the proximal small intestine obtained before the initiation of antimicrobial treatment did not reveal the presence of any other enteropathogens.

Interestingly, antibiotic treatment of *Chlamydophila psittaci* infections may result in regression of ocular adnexal lymphomas, which are usually marginal zone B-cell lymphomas of MALT type (Ferreri et al. 2012; Senff et al. 2008). Ferreri et al. (2005) reported that therapy with doxycycline was followed by lymphoma regression in 50% of patients, including those resistant to standard therapy.

Zhang et al. (2021) showed that the microbiotic community of patients with gastritis is more similar to that found in patients with gastric cancer than those present in healthy controls. Furthermore, chemotherapy reduced bacteria levels in gastric cancer patients by more than half: 14 genera were decreased, including 12, which were enriched in gastric cancer group in relation to healthy controls. Importantly, this study associated *Lactobacillus* and *Megasphaera* with gastric cancer.

### Pancreatic Cancer

Pancreatic cancer is the fourth major cause of cancer-related death in the USA, with the vast majority of patients (93%) dying within 5 years of initial diagnosis (Fan et al. 2018). Multiple factors including oral, GI, and intrapancreatic microbiota are likely to be involved in pancreatic carcinogenesis and may influence response to therapy (Wei et al. 2019). Poor oral health and related local microbiota changes, such as lower proportions of *Neisseria elongata, Streptococcus mitis, and Fusobacterium*, seem to be a risk factor for pancreatic ductal adenocarcinoma (PDAC) (Nagano et al. 2019; Olson et al. 2017; Wei et al. 2019). On the other hand, genus *Leptotrichia* and its phylum *Fusobacteria* were associated with a lowered risk of pancreatic cancer (Fan et al. 2018; Nagano et al. 2019).

*P. gingivalis* infection was reported to increase the risk of PDAC development by 59% (Fan et al. 2018). Studies of blood antibodies against *P. gingivalis* ATTC 53978 revealed higher levels in patients with PDAC than in healthy controls (Michaud et al. 2013) and levels > 200 ng/ml were associated with a twofold increase in the risk of pancreatic cancer suggesting that they may serve as a marker of increased PDAC risk (Michaud et al. 2013; Wei et al. 2019). Gnanasekaran et al. (2020) showed in vivo that pancreatic tumor cell proliferation is enhanced by *P. gingivalis* independently of Toll-like receptor (TLR)2. Furthermore, the authors found that hypoxia, a dominant feature of the PDAC microenvironment, greatly enhances *P. gingivalis* intracellular survival (Gnanasekaran et al. 2020).

Such major periodontitis-causing pathogens as *P. gingivalis, Treponema denticola,* and *Tannerella forsythia* secrete peptidyl-arginine deiminase enzymes, which degrade arginine and can can cause p53 and K-ras point mutations associated with poor prognosis in PDAC patients (Wei et al. 2019). Moreover, *P. gingivalis* can negatively affect leukocyte-mediated bacteria killing mechanisms by inhibition of IL-8 secretion (local chemokine paralysis), complement activity and TLR4 activation. These effects facilitate local inflammatory responses that contribute to progression of periodontitis (Tribble et al. 2013).

Immune activation and bacteria-related inflammation could play some role in pancreatic tumorigenesis by increasing proinflammatory cells and cytokines, oxidative stress damaging DNA, and altering energy metabolism. Consequently, bacterial infections could result in molecular alterations promoting tumor growth and metastases (Wei et al. 2019). In animal models pancreatitis and the previously mentioned *K-ras* gene mutations were found to be necessary for the development of pancreatic intraepithelial neoplasia and invasive carcinoma (Guerra et al. 2007).

PDAC incidence in humans was reported to be higher in the presence of *H. pylori*, *Enterobacter*, and *Enterococcus* spp. GI infections (Wei et al. 2019). Although in PDAC *H. pylori* DNA is detected neither in pancreatic juice nor tissue (Jesnowski et al. 2010), it could exert its negative effects indirectly by facilitating inflammation (Wei et al. 2019). Maekawa et al. (2018) showed that *Enterobacter* and *Enterococcus spp.* were the predominant bacteria in bile of PDAC patients, and the levels of antibodies against *Enterococcus faecalis* capsular polysaccharide were increased in serum supporting the concept of a causal relationship between these bacteria and PDAC.

### Hepatocellular Carcinoma

Hepatocellular carcinoma (HCC) is currently the third leading cause of cancer-related death worldwide (El-Serag and Kanwal 2014). Although HCC is closely related to chronic infection with hepatitis B virus and hepatitis C virus as well as to chronic liver damage (Dhifallah et al. 2020), a general gut microbiota dysbiosis (Ni et al. 2019) and an increase in 13 specific genera including *Gemmiger* and *Parabacteroides* were found in its early stages (Ren et al. 2019).

Mechanisms contributing to bacterial liver carcinogenesis are likely to be indirect and include the following : (1) increased intestinal permeability caused by alterations in the tight junctions between enterocytes allowing for the inflow of such harmful substances as LPS into portal blood. (2) Modification of specific receptor activity which consequently allows for passage of microbial metabolites into circulation. (3) Increased secretion of biochemically active factors (e.g. upregulation of transcription of various cytokines and receptors associated with innate and Th1-type adaptive immunity by *Helicobacter hepaticus)* (Fox et al. 2010).

Gut microbiota dysbiosis, which is common in HCC, increases LPS blood levels and may consequently lead to further liver damage (Ma et al. 2018; Yu and Schwabe 2017). Moreover, using antibiotics in rats to reduce LPS levels or genetic ablation of its receptor TLR4 prevented excessive tumor growth and multiplicity (Yu et al. 2010). TLR4 on both parenchymal (hepatocytes) and nonparenchymal cells such as Kupffer cells recognizes endotoxin and activates transcription factors that initiate innate immune response (Yu et al. 2010). The latter cells are the main target of LPS, which may lead to hepatic damage by producing proinflammatory cytokines (e.g. TNF-α and IL-6) (Anderson and Van Itallie 1995).

While there is no generally agreed upon marker of dysbiosis, a novel integrated index called degree of dysbiosis (*D*_*dys*_) was proposed by Ni et al (2019). *D*_*dys*_ is based on the relative abundance of seven protective bacteria commonly decreased in patients with chronic liver diseases (*Anaerostipes, Bifidobacterium, Coprococcus, Faecalibacterium, Lactobacillus*, *Oscillibacter*, and *Phascolarctobacterium*) and 13 potentially harmful bacteria, which are often increased in these patients (*Akkermansia, Bacteroides, Clostridium, Dorea, Escherichia, Fusobacterium, Haemophilus, Helicobacter, Klebsiella, Prevotella, Ruminococcus, Streptococcus*, and *Veillonella*) (Fox et al. 2010; Malaguarnera et al. 2010; Ren et al. 2019). In the study by Ni et al. (2019) *D*_*dys*_ were higher in patients with primary HCC when compared to healthy controls, and increased in parallel to HCC progression. However, this parameter could not reliably determine cancer stage in individual patients (Ni et al. 2019).

### Colorectal Cancer

Colorectal cancer (CRC) is the second leading cause of cancer death in the USA (Baxter et al. 2014) and colon microbiota changes could be responsible for up to 15% of all cases (Nagano et al. 2019; Parkin 2006) However, no specific bacterial species was identified as a definite CRC risk factor (Zhang et al. 2019a). CRC patients were reported to have four-fold decrease of *Eubacterium* in their gut (Balamurugan et al. 2008), which negatively affects the production of butyric acid (Zhang et al. 2019a). This short-chain fatty acid provides energy for colonic epithelial cells, regulates cellular gene expression, and could play an important role in the protection from cancer development (Scharlau et al. 2009). A significantly increased diversity of *Clostridium leptum* and *C. coccoides* was found in CRC and polypectomized patients (Scanlan et al. 2008).

Moore and Moore (1995) showed that *Bacteroides vulgatus*, *Bacteroides stercoris, Bifidobacterium longum,* and *Bifidobacterium angulatum* were associated with high risk of colon cancer and total concentrations of bifidobacteria correlated with higher risk of colon cancer. On the other hand, these authors found that Lactobacillus sp. and *Eubacterium aerofaciens* were associated with lowered odds of colon cancer oncogenesis.

Patients with CRC were found to have a significant elevation of the Bacteroides/Prevotella population and elevated number of IL-17 producing cells in the mucosa compared to control subjects with normal colonoscopy findings (Sobhani et al. 2011). Moreover, gut microbiota disturbances varied depending on their disease stage. The authors (Sobhani et al. 2011) speculated that the levels of Bacteroides/Prevotella were not the result of carcinogenesis, since they did not correlate with tumor size. *B. fragilis* was proposed as a likely carcinogen because of its ability to produce metalloprotease in CRC patients (Sears et al. 2008) and as mucosal regulatory T-cell responses inductor in experimental models (Ivanov et al. 2008; Mazmanian et al. 2008). Moreover, it was suggested that the immune response in colon cancer tissue characterized by IL-17 overexpression exacerbating the disease is due to Bacteroides (Sobhani et al. 2011; Wu et al. 2009).

*Fusobacterium nucleatum*, *B. fragilis*, and *E. coli* expressing polyketide synthase (*pks)* are also likely to be important for colonic tumorigenesis (Garrett 2019). *F. nucleatum* was reported to be abundant in CRC tissue in patients with post-chemotherapy recurrence (Yu et al. 2017) and was found to promote transformed cells proliferation in vitro (Bullman et al. 2017), likely due to the action of its adhesin and FadA activity, which bind to E-cadherin on the surface of epithelial cells and play an important role in malignant cell transformation and cancer progression (Pecina-Slaus 2003). Binding of FadA to E-cadherin activates Wnt/*β*-catenin signalling and consequently nuclear translocation of *β*-catenin and overexpression of inflammatory genes and oncogenes c-Myc and Cyclin D1 (Rubinstein et al. 2013). *F. nucleatum* binds preferentially to cancerous cells and is aided by Annexin A1, which is expressed in proliferating CRC cells. While *F. nucleatum* is detected in both colorectal adenoma and adenocarcinoma, the FadA gene levels are significantly higher in the latter (Rubinstein et al. 2019). However, *Fusobacterium* is detected in less than half of all GI adenoma cases (Baxter et al. 2014).

**Fig. 1:**
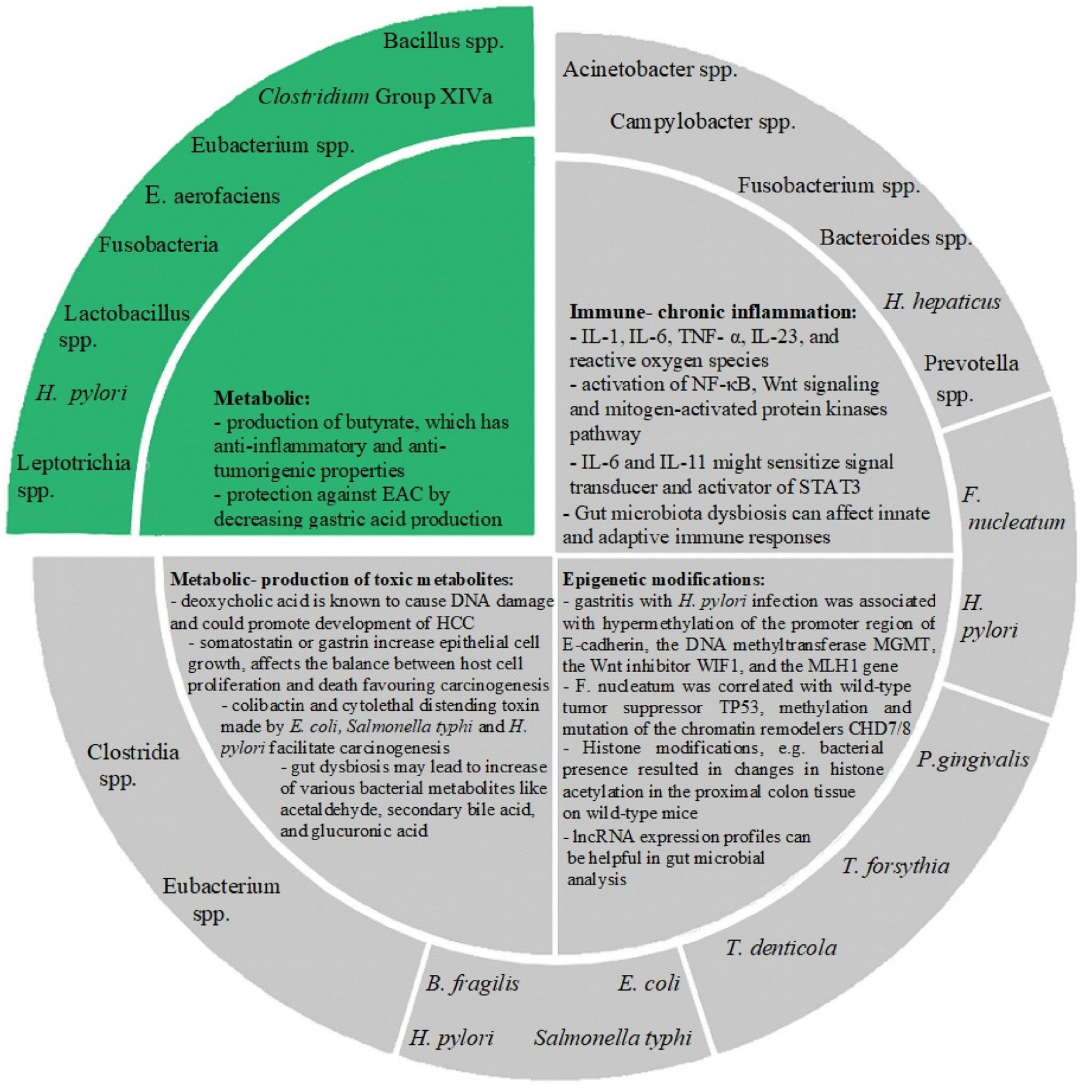
Mechanisms by which gastrointestinal (GI) microbiota could contribute to GI cancers. The mechanisms and bacteria driving oncogenesis are marked in grey, while protective mechanisms and bacteria are in green. Note that some bacteria have more than one mechanism of action. *E. aerofaciens- Eubacterium aerofaciens*, *H. pylori- Helicobacter pylori, H. hepaticus- Helicobacter hepaticus, F. nucleatum- Fusobacterium nucleatum, P. gingivalis- Porphyromonas gingivalis, T. forsythia- Tannerella forsythia, T. denticola- Treponema denticola, E. coli- Escherichia coli, B. fragilis- Bacteroides fragilis,* EAC- esophageal adenocarcinoma*,* IL-1- interleukin- 1, IL-6- interleukin- 6, IL-23- interleukin- 23, TNF- α- tumor necrosis factor-α, NF-κB- nuclear factor kappa-light-chain-enhancer of activated B cells, lncRNA- long non-coding RNA, HCC- hepatocellular carcinoma

Interestingly, Fusobacterium was found to promote chemoresistance in CRC by increasing the production of inflammatory cytokines and by modulating tumor immune microenvironment (Yu et al. 2017). In preclinical models, tumors with high figures of *F. nucleatum* exhibit increased resistance to such commonly used chemotherapeutic as oxaliplatin. *F. nucleatum* was found to activate autophagy (a cellular recycling process which affects cell survival) through TLR4 expression on CRC cells (Garrett 2019) (Fig. 1).

Kanazawa et al. (1996) studied 13 males, who previously underwent surgery for sigmoid colon cancer and later developed new second or third colonic epithelial neoplasia, and compared them to fourteen healthy controls. He found increased levels of succinic, lactic, propionic, and isovaleric acids and increased fecal pH, as well as increased numbers of Clostridia and Lactobacillus (Kanazawa et al. 1996). Thus, microbiota could play an important role in colon carcinogenesis, because feces of high-risk patients is abundant in cancer promoters.

Several mechanisms have been proposed to explain the effect of bacteria on CRC development. The “alpha-bug” hypothesis assumes that bacteria induce CRC by a specific action such as the one described for *Bacteroides fragilis* toxin which decreases E-cadherin on the surfaces of epithelial cells loosening intercellular junctions and thus allowing for an increased inflow of harmful substances and antigens from the gut (Sears et al. 2008). “Driver-passenger” hypothesis assumes that some other harmful bacteria (passenger bacteria) could adapt to the environmental changes produced by the driver bacteria and promote tumor growth. Thus, the tumor environment may select for certain bacteria, which in turn drive the tumor process. Biofilm hypothesis postulates the role of the biofilm produced by the gut microbiota and involves lack of E-cadherin or activation of signal transducers and activator of transcription 3 (Nagano et al. 2019). Bacterial biofilms are carcinogenic only in the context of specific bacteria, especially Fusobacteria (Dejea et al. 2014), and bacteria demonstrating invasion and co-aggregation properties are required for the formation of tumor-promoting biofilms (Li et al. 2017). Finally, the bystander effect hypothesis emphasizes the harmful effects of microbiota-produced metabolites (Van Raay and Allen-Vercoe 2017).

Interestingly, the colonic mucosa biofilm in patients with familial adenomatous polyposis was reported to be composed mainly of *E. coli* and *B. fragilis* already at an early noncancerous stage (Dejea et al. 2018) and experimental colonization of tumor-prone mice with these bacteria resulted in an increase of IL-17 levels and DNA damage in colonic epithelium as well as in faster tumor onset and higher mortality (Dejea et al. 2018).

Based on metagenomic analyses a number of microbes have been proposed as biomarkers of CRC, but only *F. nucleatum* has been confirmed in studies involving global cohorts. However, this biomarker is not sufficiently specific and sensitive to allow for non-invasive CRC diagnosis (Chang et al. 2021). Promising results were also provided by analysis of *Streptococcus bovis*, which has been associated with colon cancer (Tjalsma et al. 2006). An immunocapture mass spectrometry analysis of *S. bovis* antigen profiles could distinguish 11 out of 12 colon cancer patients from 8 control subjects, whereas *E. coli* antigen profiles were not useful (Tjalsma et al. 2006). Furthermore, these *S. bovis* antigen profiles were also detected in patients with polyps, suggesting that this infection occurs in the early stage of carcinogenesis. This could be a promising diagnostic tool for the early detection of human colon cancer (Tjalsma et al. 2006).

Probiotics show promise as agents of host–microbiome modulation therapies for several diseases, including CRC (Torres-Maravilla et al. 2021). It has been proposed that probiotics may minimize the development and progression of CRC by mitigating the aggressiveness of tumors. Bacillus and Saccharomyces and next-generation probiotics are currently tested in clinical trials (Torres-Maravilla et al. 2021).

Although the overwhelming majority of studies support the role of the microbiome in cancer, some authors did not find significant associations. For example, in the study by Olson et al. (2017) there were no statistically significant differences in oral bacterial composition among patients with PDAC, patients with intraductal papillary mucinous neoplasms and healthy controls.

## Conclusions

A large number of studies have been devoted in recent years to the analysis of the role of microbiota in GI oncogenesis. New treatments such as fecal transplantation, phage-based therapy, antibiotics, and probiotics therapies are currently tested in clinical settings to detect, prevent or improve the clinical course of various cancers. There is also a concerted effort to develop a fast, non-invasive, sensitive, and specific cancer detection test. However, these novel treatment interventions and diagnostic tests require validation in rigorous clinical trials before they could enter clinical practice.

## Data Availability

Not applicable.
